# Multifactorial causation of early onset colorectal cancer

**DOI:** 10.7150/jca.63676

**Published:** 2021-09-24

**Authors:** Darina Lazarova, Michael Bordonaro

**Affiliations:** Department of Medical Education, Geisinger Commonwealth School of Medicine, 525 Pine Street, Scranton, PA 18509, USA.

**Keywords:** colon cancer, obesity, mutations, body mass index, metabolic syndrome, cell signaling, febrile episodes, stem cells, antipyretics

## Abstract

The multiple-hit hypothesis of cancer, including colorectal cancer (CRC), states that neoplastic development requires a sequence of mutations and epigenetic changes in driver genes. We have previously proposed that obesity increases CRC risk by supporting neoplastic development through adipokine-induced signaling, and this proliferative signaling substitutes for specific driver gene mutations. In support of this hypothesis, analyses of The Cancer Genome Atlas (TCGA) mutation data have revealed that obese patients with microsatellite stable CRC exhibit fewer driver gene mutations than CRC patients with normal body mass index. The lower number of driver gene mutations required for cancer development may shorten the neoplastic process and lead to an early onset of CRC. Therefore, obesity could be one factor explaining the rise of CRC incidence among younger individuals (< 50 years of age); furthermore, early onset CRC has been associated with the increasing incidence of metabolic syndrome and obesity in this age group. However, CRC incidence among older individuals (> 50 years of age) is stable or declining, despite the high rates of metabolic syndrome and obesity in this age group. In search for explanations of this phenomenon, we discuss several factors that may contribute to the divergent CRC incidence trends in populations under, and above, the age of 50, despite the rising levels of metabolic syndrome and obesity across all ages. First, older individuals with metabolic dysregulation are more frequently on maintenance medications, such as aspirin, β-blockers, lipid-lowering drugs, ACE inhibitors, metformin, etc., compared to younger individuals. Such treatments may suppress specific adipokine-induced proliferative signaling pathways, and therefore counteract and slow down neoplastic development in medicated overweight/obese individuals. Second, in the past decades, the incidence of infectious diseases accompanied by febrile episodes has been decreasing and the use of antipyretics increasing. Compared to normal cells, neoplastic cells are more sensitive to high body temperature; therefore, the decreased number of febrile episodes in childhood and adolescence may contribute to increased cancer incidence before the age of 50. Third, obesity at younger age may expand the stem cell compartment. An increased number of intestinal stem cells and stem cell divisions translates into a higher probability of sporadic mutations in the stem cells, and therefore, a greater chance of neoplasia. In conclusion, we hypothesize that early onset CRC has multifactorial causation and the proposed associations could be examined through analyses of existing data.

## Introduction

In the United States of America (USA), the incidence of colorectal cancer (CRC) has been increasing among younger individuals (<50 years of age); however, the incidence for CRC among older individuals (>50 years of age) has been stable or declining 1-3. The median age for CRC diagnoses has shifted from 72 years in the early 2000s to a more recent figure of 66 years 4. Compared to adults born circa 1950, those born circa 1990 have doubled the risk of colon cancer 5. Colon cancer incidence rates has increased by 1.0% to 2.4% annually since the mid-1980s in adults aged 20 to 39 years and by 0.5% to 1.3% since the mid-1990s in adults aged 40 to 54 years; rectal cancer incidence rates have been increasing even faster in these age groups 1. In contrast, in adults age 55 years and older, incidence rates for colon cancer declined since the mid-1980s, and since 1974 for rectal cancer. From 1989-1990 to 2012-2013, rectal cancer incidence rates in adults aged 50 to 54 years went from half those in adults aged 55 to 59 to equivalent (24.7 vs 24.5 per 100,000 persons), and the proportion of rectal cancer diagnosed in adults under the age of 55 doubled from 14.6% to 29.2%. This is an international trend, as CRC incidence has been increasing among young adults in high-income countries 1.

Increased diagnostic screening cannot account for the rising incidence of CRC among the young, since the highest rate of disease increase is among adults 20 to 30 years old who are not typically screened 6. The increasing incidence of early onset (under the age of 50) CRC (EOCRC) has instead been associated with obesity and metabolic dysregulation. Furthermore, higher body mass index (BMI) has been associated with a specific type of CRC that contains fewer malfunctioning stem-like cells, which may fail to differentiate into glandular epithelial cells, and depends on an excess energy balance 7.

Obesity is a significant problem in developed nations such as the USA 8, and it has been linked to increased risk not only for CRC, but other types of cancer as well 9-13. Obesity is frequently associated with metabolic syndrome, which is defined as the occurrence of at least three of the following: abdominal obesity (waist circumference of greater than 40 inches in men, and greater than 35 inches in women), triglyceride level of 150 milligrams per deciliter of blood (mg/dL) or greater, HDL cholesterol of less than 40 mg/dL in men or less than 50 mg/dL in women, systolic blood pressure of 130 millimeters of mercury (mm Hg) or greater, or diastolic blood pressure of 85 mm Hg or greater, and fasting glucose of 100 mg/dL or greater 14. The prevalence of metabolic syndrome and obesity increase with age, with the highest risk observed for individuals over the age of 60 15. However, in parallel with the rise of obesity, metabolic syndrome has also been increasing in the pediatric population 16.

Meta-analyses have established a significant association between the presence of metabolic syndrome and colorectal neoplasia. Overall, metabolic syndrome associated with a 34% increase in the risk of colorectal neoplasia in both genders, and the association was significant when analyses were applied to both adenoma and cancer 17. Another study based on meta-analyses confirmed the association between metabolic syndrome and an increased risk of CRC incidence and mortality in both men (RR: 1.33, 95 % CI: 1.18-1.50, and 1.36, 1.25-1.48, respectively) and women (RR: 1.41, 1.18-1.70, and 1.16, 1.03-1.30, respectively) 18. Furthermore, metabolic syndrome has been specifically associated with EOCRC. Thus, a nested case-control study of individuals aged 18 to 64 established that metabolic syndrome associated with higher risk of EOCRC (n=4673; multivariable adjusted OR 1.25; 95% CI: 1.09 to 1.43), similar to CRC risk diagnosed at age 50 to 64 (n=14 928; OR 1.21; 95% CI: 1.15 to 1.27) 19. In the same study, compared to individuals without a metabolic comorbid condition, the individuals with one, two, or at least three such conditions were observed to have a 9%, 12%, and 31% higher risk of EOCRC, respectively; however, among individuals at age 50 to 64, there was no association for one or two metabolic comorbid conditions and CRC risk. Numerous other studies have confirmed the association between metabolic syndrome and risk of CRC 20-25. In summary, despite that overweight/obesity in the USA is increasing across all age groups, CRC incidence is rising among those under the age of 50 and is stable or declining among those over the age of 50 1-3,26,27.

We have previously proposed a possible mechanism whereby obesity promotes neoplastic development in the colon and rectum 28. According to the multiple hit hypothesis of cancer, a sequence of mutations and epigenetic changes in driver genes is required for the neoplastic development. We have posited that obesity contributes to increased risk of CRC by providing sustained proliferative signaling that allows for neoplastic development despite a relatively low number of driver gene mutations. This hypothesis is supported by analyses of The Cancer Genome Atlas (TCGA) data. Thus, obese patients with microsatellite stable (MSS) CRC exhibit fewer mutations in specific driver genes compared to patients with normal BMI 28. Excessive adipose tissue could support neoplasia-promoting MEK/ERK and other cell signaling cascades, and therefore, may account for the lower number of *KRAS* and *PTEN* mutations in colonic cancer of obese individuals 28. Since the accumulation of mutations requires time, the relatively lower number of driver gene mutations required for the neoplastic development in overweight/obese individuals would translate into an EOCRC. Therefore, the increasing rates of obesity/metabolic syndrome among children, adolescents, and young adults 29 may be in part responsible for the increased incidence of EOCRC and other types of cancer 1-3, 26,27.

In the present paper, we expand our discussion by proposing additional factors that account for EOCRC. We hypothesize that the increasing incidence of CRC in younger individuals (under the age of 50) and the decreasing incidence of CRC among older individuals (above the age of 50) is due to (1) the more frequent use of maintenance medications for metabolic dysregulation/obesity in individuals above age of 50, (2) the increased use of antipyretics and decreased incidence of febrile episodes in children and adolescents, and (3) the expansion of the intestinal stem cell niche compartment due to obesity.

The rationale for considering these three hypotheses is that there are (a) age-related epidemiological correlations between CRC incidence, obesity, the use of maintenance medication for obesity-related metabolic disorders, and frequency of febrile episodes; and (b) established physiological mechanisms by which obesity, treatment for metabolic disorders, and frequency of febrile episodes contribute to carcinogenesis. Hence, it is reasonable to determine whether these physiological mechanisms contribute in a causal manner to the association between CRC and the aforementioned epidemiological trends. The hypotheses presented here suggest that obesity and its consequences, treatment for those consequences, as well as frequency of febrile episodes contribute to age-related CRC risk in a causal manner via known physiological mechanisms. We then propose tests of these hypotheses to determine if the proposed associations are indeed causally linked. Thus, the proposed associations of these factors with the increased rates of EOCRC might be amenable to analyses of existing data, as well as experimental studies.

## Treatment of metabolic dysregulation and CRC incidence among age groups

**Figure 1:**
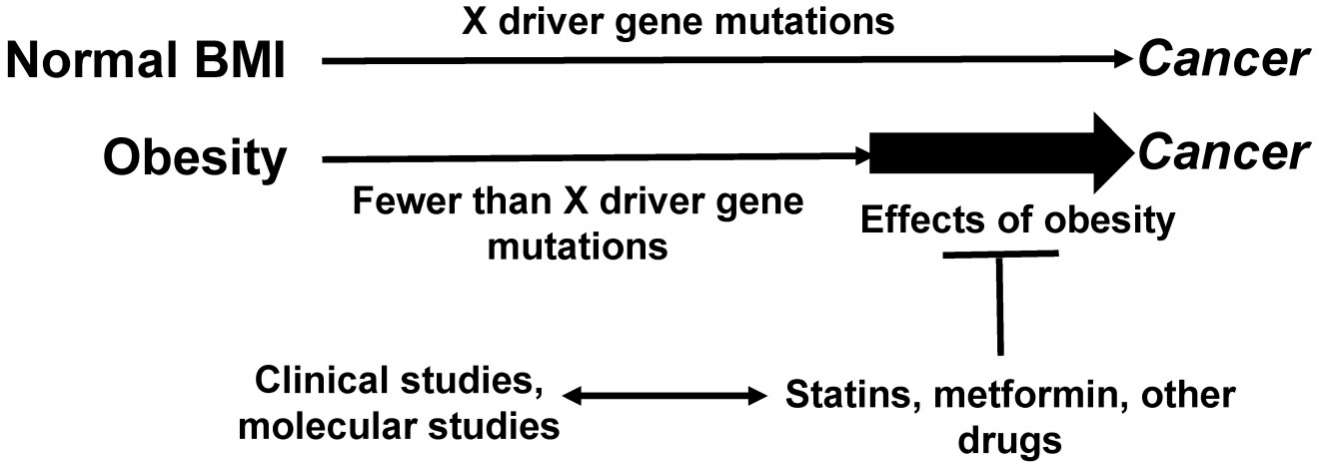
** Relationship between maintenance medications, driver gene mutations, and CRC risk.** Obese CRC patients exhibit fewer driver gene mutations compared to CRC patients with normal BMI 28. By augmenting the levels of proliferative signaling pathways, obesity may allow for a lower threshold of fewer driver gene mutations required for neoplastic development. Maintenance medications, typically more frequently prescribed to older patients with various metabolic conditions, might interfere with the effects of obesity on tumorigenesis, and this would contribute to the decreasing incidence of CRC among older individuals. Clinical studies can address this hypothesis; molecular studies could elucidate the mechanisms whereby maintenance medications interfere with the obesity-fueled neoplastic development. “X” represents the number of driver gene mutations and other changes (such as epigenetic) required for cancer development.

We posit that the pharmacological interventions for metabolic dysregulation decrease the stimulatory effects of adipose tissue on neoplastic development (Fig. 1). Such medications are frequently prescribed later in life, as the detrimental effects of metabolic syndrome/obesity on the overall health status could be manifested decades after onset. Therefore, metabolic syndrome-associated conditions are not medicated as frequently in adults under the age of 50 as they are in adults over the age of 50. This difference may in part account for the decline of CRC incidence in older age groups 5 and the increasing risk of EOCRC 19.

In parallel with obesity, metabolic syndrome is increasing in prevalence. In 2017-2018, the age-adjusted prevalence of obesity in adults was 42.4%, and this prevalence was age-dependent: the obesity was 40.0% among adults aged 20 to 39 years, 44.8% among adults aged 40 to 59 years, and 42.8% among adults aged 60 and older 30. Concomitantly, the prevalence of metabolic syndrome has been increasing and in the period 2007 to 2014, 34.3% of the adults were diagnosed; this prevalence increased with age, from 19.3% among people 20 to 39 years of age to 37.7% for people 40 to 59 years of age and 54.9% among people ≥60 years of age in the period 2007 to 2014 31. The same trends were observed among the young US population: in 2020, 19.3% of US young people, ages 2 to 19, were obese, compared to 5.5% in the mid-1970s 30,32. The prevalence of metabolic syndrome in obese youth was 19% to 35%, compared with less than 2% in the normal-weight groups 33. A meta-analysis of the next age group, that of young adults 18 to 30 years of age, revealed prevalence of metabolic syndrome of 4.8% to 7%, depending on the definition used 34.

The most frequent pharmacological interventions for metabolic syndrome and its health consequences are aspirin, β-blockers, lipid-lowering therapy, and angiotensin-converting enzyme (ACE) inhibitors, and these medications are not proportionally prescribed to individuals under, and above, the age of 50. Statin use in the general population increased from 4% in 1992 to 41% in 2008 35. During 2003 to 2012, the percentage of adults aged ≥40 years who had used a cholesterol-lowering medication in the past 30 days increased from 20% to 28% 36. The antihypertensive drug use also increased from 53% in 1992 to 74% in 2008 35. In 2005 to 2010, 30.9% of US adults over 80 years of age were taking at least three classes of antihypertensive medication 37. The increased use of statins and antihypertensive drugs has not been proportional among the different age groups diagnosed with metabolic dysregulations; thus, in 2011 to 2012, antihypertensive medication use was lowest for individuals aged 18 to 39 years (44.5%) compared with those aged 40 to 59 years (73.7%) and those aged over 60 years of age (82.2%) 36. The use of prescription cholesterol-lowering medications has also increased with age, and adults aged 40 to 64 who reported having health insurance or prescription medication coverage were more likely to take cholesterol-lowering medications compared to other age groups 36.

In addition to the health consequences of metabolic syndrome being manifested later in life and addressed by prescribed medications at that time, there is also lower adherence to prescribed medications among the younger population, and this is in part due to inadequate health insurance. There are compelling statistics on adherence to prescribed medications for diabetes, the incidence of which has been increasing for decades largely due to the increase in obesity 38. For example, among adults aged 18 to 64 with diabetes, the percentage who did not take their prescribed medication, or asked their doctor for a lower-cost medication, was highest among the uninsured; whereas, among adults aged 65 and over with diabetes, the percentage who asked their doctor for a lower-cost medication, was lowest among those with Medicare and Medicaid coverage 39. Furthermore, adults under the age of 65 with diabetes were more likely than those aged 65 and over to not take their medication as prescribed (17.9% and 7.2%, respectively) and to ask their doctor for a lower-cost medication (26.3% and 21.9%, respectively) 39. Adherence to diabetes medication is strikingly different across age groups. Compared with individuals 45 to 64 years of age, 49% of the individuals 25 to 44 years of age were less likely to be adherent with their diabetes medications; conversely, individuals aged 65 to 74 years were 27% more likely to be adherent, and those aged 75 years and above were 41% more likely to be adherent when compared with the group of 45 to 64 years 40. Considering the prevalence of diabetes and prediabetes in the USA. (34.2 million and 88 million, respectively) 32, there are great numbers of patients who do not adhere to their medication, particularly among the young. Therefore, younger patients are, for various reasons, more likely to not be on maintenance medication, and this can at least partially explain increased cancer incidence in this age group if maintenance medication can suppress neoplasia.

Statins, metformin, aspirin and other medications prescribed for metabolic dysregulation are known to reduce the risk of CRC and other forms of cancer 41-43. For example, data on the molecular effects of metformin may explain its impact of cancer risk 42. These mechanisms suggest associations between metabolic syndrome/diabetes type II and cancer cell metabolism/cell signaling. Metformin, prescribed to treat metabolic syndrome/diabetes type II, reduces cancer risk, and data suggest that this activity is at least in part due to the ability of metformin to affect blood glucose 42. Thus, loss of 5-hydroxymethylcytosine (5hmC) is a known epigenetic characteristic of cancer; 5hmC can be produced by oxidation of 5mC by ten-eleven translocation (TET) enzymes, including the TET2 tumor suppressor 42. TET2 is phosphorylated and stabilized by 5' AMP-activated protein kinase (AMPK); however, this phosphorylation is inhibited by high glucose levels, thus decreasing the levels of TET2, and leading to lower levels of 5hmC 42. Metformin “protects” the ability of AMPK to phosphorylate and stabilize TET2, leading to higher levels of 5hmC, and this effect is likely protective against cancer 42. This is just one metabolic link between diabetes and cancer risk, and a proposed mechanism for the protective action of metformin against diabetes-associated cancers. Considering that metabolic syndrome and type II diabetes are strongly linked to obesity 32, the association between metformin and cancer risk demonstrates how increased use of maintenance medication among older obese individuals can reduce cancer incidence compared to obese younger individuals.

## Testing the hypothesis: Treatment of metabolic dysregulation

The hypothesis that medications prescribed for metabolic conditions such as hypertension, high cholesterol, high blood glucose, and insulin resistance counteract the proliferative effect of adipose tissue on neoplastic cells in the colon and rectum, and this protective effect against CRC is mostly observed in individuals older than 50 since they are more frequently on such medications, could be tested by comparative analyses of CRC incidence between individuals medicated for metabolic dysregulation and individuals who are not medicated for the same metabolic conditions. This type of analyses would be performed separately for individuals under the age of 50 and those older than 50. It is unknown how long the neoplastic process for EOCRC is; therefore, in the analyses, to account for this unknown factor, it would be reasonable to use a ratio of the years of metabolic dysregulation (or overweight/obesity) during which an individual was medicated *versus* the years of metabolic dysregulation (or overweight/obesity) during which the individual was not medicated. A comprehensive study should take into account metabolic syndrome interventions such as metformin, antidiabetics (e.g., biguanides), antidiabetics (e.g., thiazolidinediones), lipid-lowering agents (e.g., statins), ACE Inhibitors, angiotensin II receptor blockers, lipid-lowering agents (e.g., non-statins), antiplatelet agents, etc. The clinical studies could be both retrospective and prospective, with the appropriate control on variables and confounding factors. Any metabolic syndrome non-pharmacological interventions such as dietary changes, nutritional counseling associated with change in BMI should be taken into account. It is expected that among individuals with metabolic dysregulation, those who have the highest ratios of medicated to non-medicated years would exhibit the lowest odds ratios of CRC incidence.

**Figure 2:**
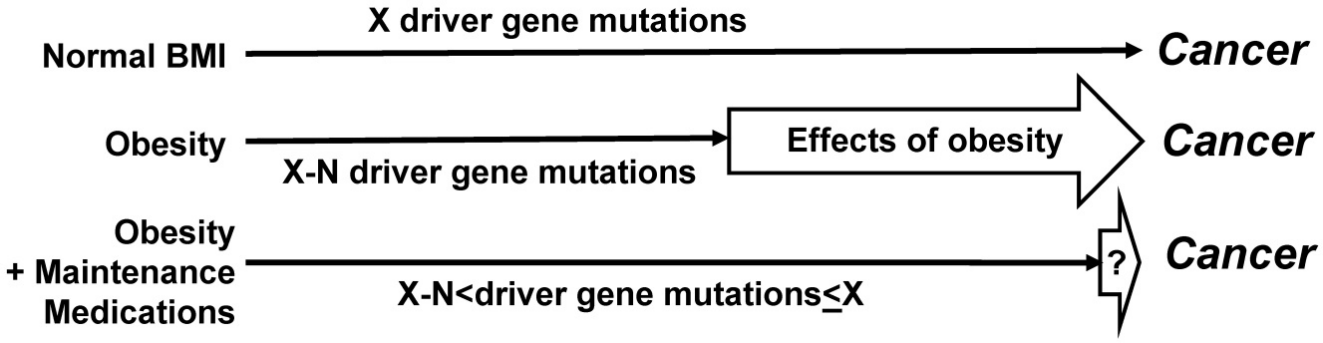
** Effects of maintenance medications on driver gene mutations**. Compared to not medicated CRC patients with metabolic dysregulation, medicated CRC patients with metabolic dysregulation are expected to have a greater number of driver gene mutations. Dependent upon the extent to which medications counteract the stimulatory effects of obesity on neoplastic development, the average number of driver gene mutations in medicated CRC patients with metabolic dysregulation may not differ in a statistically significant manner from the number of driver gene mutations in not medicated CRC patients without metabolic conditions. However, statistically significant lower numbers of driver gene mutations are expected in not medicated CRC patients with metabolic dysregulation. “N” is the difference in number of driver gene mutations (“X”) required for cancer development.

For the molecular studies, it would be optimal to collect mutational data, and in particular, to determine the driver gene mutations. Distinguishing driver from passenger genes could be based upon published data 44, and the continuously updated mutation data reported by the Broad Institute of Harvard and MIT (http://www.tumorportal.org/). We expect that CRC patients with low ratio of medicated to non-medicated years of metabolic dysregulation would have fewer driver gene mutations than CRC patients with normal BMI/absence of metabolic dysregulation who are not on medications. Another expectation is that patients with metabolic condition(s) who develop CRC despite a high ratio of medicated vs non-medicated years of metabolic dysregulation would exhibit a greater number of driver gene mutations (and/or epigenetic changes) than patients with the same metabolic conditions who were not on such medication(s) or had a low ratio of medicated vs non-medicated years of metabolic dysregulation (Fig. 2). Such finding could be explained by the medications counteracting the ability of obesity/metabolic dysfunction-associated adipokines to substitute for driver gene mutations in the neoplastic process. Therefore, any CRCs that do develop in medicated individuals would require more mutations to “compensate” for the loss of proliferative signaling provided by dysregulated metabolism.

We expect that a comparison of medicated CRC patients with metabolic conditions to non-medicated CRC patients with normal BMI and free of metabolic conditions would not reveal statistically significant differences in numbers of driver gene mutations (Fig. 2). If specific pharmacological agents for metabolic dysregulation reduce CRC risk in the clinical studies, then analyses on the molecular mechanisms might reveal the relevant targets of the agents that influence cell metabolism, signaling, and inflammation in metabolic dysregulation - associated CRC development. Molecular studies could include cell culture and organoids (*in vitro*) and mouse models (*in vivo*).

## Effect of obesity on the intestinal stem cell niche

We propose that obesity-induced expansion of the intestinal stem cell niche provides a broad base for sporadic mutations, some of which may contribute to CRC. Obesity-induced expansion of the stem cell niche is expected to be more prominent and effective at younger age than at older age. Thus, it has been reported that the regenerative potential of the intestinal stem cells (ISCs) declines with age 45. Compared to ISCs in young mice, the ISCs in old mice have a lower regenerative capacity, leading to fewer Lgr5^+^ cell-derived clones in the older animals 46.

The ISCs reside at the base of the intestinal crypts, either in the +4 position, counting from the bottom of a crypt, or at the bottom of the crypts, where the crypt base columnar cells (CBCs) are located 47. The stem cells at the +4 position are quiescent; whereas, the CBCs are continuously activated by signals from stromal cells and, therefore, regenerate themselves and give rise to progenitor cells. In mice, CBC ISCs positive for the expression of *Lgr5* are the cells that originate adenomas after a loss of function of the Apc tumor suppressor gene 48.

A vast body of evidence supports the view that nutrient availability and nutrient overload increase the number of total and proliferative ISCs 49,50. The higher the number of stem cells, and the more cell divisions they go through, increase the chance for sporadic mutations and, therefore, for neoplastic development. Studies performed in *Drosophila* have demonstrated that fasting decreased the number of ISCs; however, refeeding restored this number 51. Nutrient availability in *Drosophila* led to accelerated division of ISCs and predominance of symmetric division that increased the number of stem cells 52. Interestingly, in mammals, long-term calorie restriction increased the numbers and proliferation of ISCs 53. However, our hypothesis is not dependent upon possible compensatory mechanisms promoted by long-term calorie restriction, but it is rather based upon the observation that diet-induced obesity increases ISC numbers in mammalian models. Therefore, highly relevant to our hypothesis is the finding that in a mouse model, diet-induced obesity increased the number of ISCs and the hyperproliferation of ISCs *in vivo* 54. Thus, a hypercaloric load increased the number of total and proliferating (S-phase) ISCs, and these changes were associated with elevated plasma insulin and IGF1. Another study in mice confirmed that high-fat diet-induced obesity, independent of the high-fat diet (*i.e*., independent of the type of diet), increased the number of ISCs *in vivo* 55. These data suggest that it is obesity, rather than any specific diet or calorie restriction, which increases the number of ISCs. Mechanistic studies have demonstrated that in the mammalian intestine, diet-induced obesity increased the number and function of *Lgr5*^+^ ISCs by inducing peroxisome proliferator-activated receptor delta, and the enforced PPAR-delta signaling contributed to tumorigenicity *in vivo* upon loss of the tumor suppressor Apc 56.

The obesity-associated milieu and the stromal cells in the stem cell niche also contribute to the neoplastic process in the colon and rectum. Recently, it was reported that in the intestinal stem cell niche a subset of intestinal stromal cells augment WNT signaling and maintain the LGR5^+^ intestinal stem cells 57. When compared to adipose-derived stromal/stem cells isolated from lean individuals (BMI < 25), the stromal cells from obese individuals (BMI > 30) expressed higher levels of carcinoma-associated fibroblast markers, and in co-culture with stromal cells from obese individuals, cancer cells exhibited higher proliferation, an invasive phenotype, and enhanced the expression of pro-tumorigenic factors 58.

## Testing the hypothesis: increased number of intestinal stem cells in obesity

How could we test the hypothesis that obesity differentially expands the regenerative potential of ISCs in old *versus* young individuals? The possibility that obesity impacts the proliferative potential of human ISCs is not easy to test; however, there are already experimental methodologies that suggest the feasibility of indirect research approaches: (1) actively cycling ISCs (CBCs) can be identified based upon several markers, including the expression of LGR5 59,60, and (2) isolation of human ISCs from whole tissue or biopsies has been described, and such cells have been assessed in three-dimensional organoids (“colonoids”) 61. For example, the effects of increased levels of insulin and insulin-like growth factor-1 (IGF-1) in obesity have been investigated in primary intestinal epithelial crypts isolated from obese humans. The study results indicate that in obesity, high levels of insulin and IGF-1 enhance intestinal epithelial crypt proliferation through PI3K/AKT, without involvement of the ERK signaling pathway 62. To address, at least in part, our hypothesis, similar studies could be carried out with organoids derived from young *versus* old individuals. Consistent with our hypothesis, we expect that the exposure to adipokines would more effectively enhance the proliferative potential of “young” colonoids compared to that of “old” colonoids.

## Decreased number of febrile episodes and use of antipyretics

**Figure 3:**
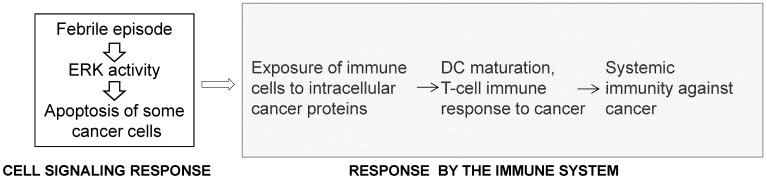
The response to high body temperature consists of two steps: a signaling response at the level of the mutant cell, and an immune response at the level of the organism. We propose that high body temperature that accompanies acute inflammations suppresses neoplastic development through the sustained hyperinduction of mutation-deregulated pathways such as RAS/MEK/ERK signaling, and the subsequent induction of apoptosis.

The inverse association between acute infections and cancer is supported by epidemiological data, recorded bacterial treatments of cancer patients, and by *in vivo* experimental work 63,64. In 1874 England, Dr. Campbell De Morgan presented evidence that, in some cases, cancer regresses after infection and, in particular, after the onset of tuberculosis 65. In 1890, during a review of records in Memorial Hospital, New York, Dr. William Coley found a case in which erysipelas cured a cancer patient 66. After researching this approach further, in 1891, Coley replicated this outcome by utilizing *Streptococcus pyogenes* and inducing erysipelas in a patient with sarcoma 67. Subsequently, Coley switched to heat-inactivated mixtures of bacteria and increased the dosage until a fever of 39 °C or higher was developed by the patients 66-69. Most of Coley's patients had late-stage cancers that did not respond to conventional treatments and yet retrospective analyses of 170 cases for which there are adequate medical records indicate five-year survival in 44% of the patients 69. In the 1960s, by not “grandfathering” Coley's treatment, the US Food and Drug Administration (FDA) stopped its application in the USA. Later, unsuccessful attempt to revive the therapy utilized a mixed bacterial vaccine (Vaccineurin); however, the treatments were of short duration and without the objective of achieving fever, which may explain the vaccine's failure, as the curative effect of acute infections is likely initiated by fever 70. The significance of developing high body temperature was confirmed in a more recent clinical trial in Germany with a mixed bacterial vaccine 71. Reminiscent of Coley's approach are the promising studies with *Clostridium novyi* reported by the group of Dr. Vogelstein at Johns Hopkins 72,73. Epidemiological analyses also support an inverse association between acute infections and cancer incidence. For example, individuals with a history of three or more infections with fever above 38.5 °C have a 40% lower risk for melanoma 74, and the anamnesis of cancer patients compared to the medical history of infectious diseases in cancer-free patients has been confirmed 75. In contrast to the inverse association between acute infections and cancer, chronic inflammations increase the risk of cancer 64. A significant difference between the two conditions is that acute inflammations lead to high fever compared to chronic inflammations that typically do not 63. Therefore, fever might be the critical anti-cancer factor, since high body temperature preferentially kills cancer cells 70, and the release of internal cancer-specific antigens from the dying mutant cells can elicit anti-cancer immune response 75 (Fig. 3). Therefore, the response to high body temperature consists of two steps: a signaling response at the level of the mutant cells, and an immune response at the level of the organism (Fig. 3). We have already provided evidence that hyperthermia induces apoptosis in CRC cells by hyper-activating ERK and WNT signaling pathways that are already deregulated by mutations in neoplastic cells 76.

We posit that in the last decades, the rate of neoplastic initiation in young adults has been increasing due to the decreasing number of febrile episodes. Febrile episode is defined as an episode of a temperature above 37.5 °C after seven consecutive days of normal body temperature of below 37.5 °C 77. The decreased number of febrile episodes among the younger generations is likely due to (1) the decreased incidence of infectious diseases in the developed countries, and (2) the increased use of antipyretics. Febrile episodes usually develop during acute infections, and fever is a response of a healthy immune system to the infectious agents. Until the last century, infections such diphtheria, diarrheal illnesses, tuberculosis, and streptococcal infections were the main causes of childhood morbidity and mortality in all countries, and still are in the developing world 78. However, infectious disease mortality has significantly decreased in the 20^th^ century 79.

In addition to the decreasing incidence of infections accompanied by febrile episodes, the use of antipyretics has been on the rise. Fever treatment includes the use of various over-the-counter antipyretics such as acetaminophen and the nonsteroidal anti-inflammatory drugs aspirin, ibuprofen, and naproxen. Fever is the most common sign of illness in pediatric practice and accounts for 19% to 30% of all visits 80,81. Younger children are specifically susceptible to febrile episodes due to their body size, low amount of subcutaneous fat, and high ratio of body surface area to weight 82. Compared to children and young adults, older adults have less exposure to antipyretics. Older adults develop fever less frequently and there is evidence that their mean body temperature decreases with age; this could be explained with the age-related deterioration of the immune system function and other physiological changes. In the elderly, fever is absent or blunted 20%-30% of the time in benign infections and is lower in severe infections such as meningitis and pneumonia 83-85. However, this lower probability for febrile episodes in old age is currently mimicked in younger generations since the “fever phobia” among parents, the advertisements for antipyretics by pharmaceutical companies, and the predominant perception among pediatricians that fever is dangerous 86,87, all have contributed to the increasing use of antipyretics. The increased use of antipyretics could decrease the effects of fever on mutant cells, and therefore, according to our hypothesis, could set the stage for an increased risk of neoplastic process.

## Testing the hypothesis: decreased number of febrile episodes

Testing the hypothesis that the rate of neoplastic initiation in young adults is rising due to the decreasing number of febrile episodes and increasing use of antipyretics is not an easy task. However, epidemiological data, electronic health records, and self-reported administration of antipyretics by caregivers may allow us to address this research question. We expect that the increased number of febrile episodes during which the individuals were not medicated with antipyretics will be associated with a decreased risk of CRC and other cancers in these individuals. In contrast, fewer febrile episodes and increased use of antipyretics would be associated with an increased risk of CRC and other types of cancer.

## Conclusion

The obesity crisis is a growing public health disaster and is associated with the increased incidence of certain types of cancer, including CRC, among younger individuals. Investigating the connection between cancer and age, metabolic status, medical history, mutations, and maintenance medication may lead to novel approaches for cancer prevention. If our hypotheses are supported by data analyses and experimentation, consideration should be made to increase cancer screening among at-risk younger patients (e.g., individuals with metabolic dysregulation who have not been medicated for the condition). However, given the scope of the crisis, immediate pharmacological interventions to reduce CRC incidence in at-risk individuals should also be considered. Knowledge of how maintenance medications prescribed for metabolic syndrome affect CRC incidence among different age groups could be of great impact. The possible link between CRC risk and decreased number of febrile episodes at younger age may lead to a debate and further education on the proper use of antipyretics. The possibility that obesity and metabolic syndrome expand the number of ISCs and stem cell divisions could be addressed through education on diet and physical activity, as well as through legislative changes that impact the current lobbies of food and drink industries. Finally, despite our effort to consider multiple factors that might contribute to the increased incidence of EOCRC, it is likely that this phenomenon is due to the convergence of additional factors that have evaded societal attention in the last several decades.

## Abbreviations

5hmC: 5-hydroxymethylcytosine
ACE: angiotensin-converting enzyme
AMPK: 5' AMP-activated protein kinase
BMI: body mass index
CBCs: crypt columnar cells
CRC: colorectal cancer
EOCRC: early onset CRC
FDA: Food and Drug Administration
ISCs: intestinal stem cells
MSI: microsatellite instability
MSS: microsatellite stable
TCGA: The Cancer Genome Atlas
TET: ten-eleven translocation
US: United States
USA: United States of America
